# Supplementary material for: Seroprevalence of SARS-CoV-2 Infection and Adherence to Preventive Measures in Cuenca, Ecuador, October 2020, a Cross-Sectional Study
Source: Int J Environ Res Public Health. 2021 Apr 27;18(9):4657. doi: 10.3390/ijerph18094657 (PMC8124135; doi:10.3390/ijerph18094657)
Supplement: Supplementary file 1 [file ijerph-18-04657-s001.zip › ijerph-1152611-supplementary.pdf]

International Journal of
Environmental Research and Public Health

MDPI

Supplementary Table

Table S1. Level of adherence to COVID-19 preventive measures and corresponding SARS-CoV-2 seroprevalence.

Variables	Response	Survey Findings, n = (2457)	SARS-CoV-2 Seropositive	Crude Odds Ratios
Observe physical distance 1.5 m rule	No, *n* (%)	182 (7.4%)	20 (11%)	
	Yes, *n* (%)	2275 (92.6%)	305 (13.4%)	1.25 (0.78–2.03)
Wear face mask when going outside	No, *n* (%)	166 (6.8%)	20 (12%)	
	Yes, *n* (%)	2291 (93.2%)	305 (13.3%)	1.12 (0.69–1.82)
Frequent hand washing	No, *n* (%)	162 (6.6%)	16 (10%)	
	Yes, *n* (%)	2295 (93.4%)	305 (13.3%)	1.42 (0.84–2.41)
Stay home when feel flu-like symptoms	No, *n* (%)	295 (12%)	32 (10.8%)	
	Yes, *n* (%)	2162 (88%)	293 (13.5%)	1.29 (0.87–1.9)
Have you been in a meeting with more than 10 people in the past 7 days?	No, *n* (%)	2079 (85%)	268 (13%)	
	Yes, *n* (%)	378 (15%)	57 (15%)	1.2 (0.88–1.64)
Have you been in a restaurant, bar, disco in the past 7 days?	No, *n* (%)	2299 (93.6%)	307 (13%)	
	Yes, *n* (%)	158 (6.4%)	18 (11%)	0.83 (0.5–1.38)
Have you attended any religious service in the past 7 days?	No, *n* (%)	2330 (95%)	306 (13%)	
	Yes, *n* (%)	127 (5%)	19 (15%)	1.16 (0.7–1.92)
Have you been to a funeral in the past 7 days?	No, *n* (%)	2401 (97.7%)	312 (13%)	
	Yes, *n* (%)	56 (2.3%)	13 (23%)	2.02 (1.08–3.81)*
Have you been in a vehicle or bus with more than 10 persons in the past 7 days?	No, *n* (%)	2094 (85%)	271 (13%)	
	Yes, *n* (%)	363 (15%)	54 (15%)	1.18 (0.86–1.61))
Have you travelled in the country in the past 14 days?	No, *n* (%)	2334 (95%)	310 (13%)	
	Yes, *n* (%)	123 (5%)	15 (12%)	0.91 (0.52–1.58)
have you travelled internationally in the past 14 days	No, *n* (%)	2454 (99.8%)	323 (13%)	
	Yes, *n* (%)	3 (0.12%)	2 (67%)	-
Physical contact with someone outside the household*	No, *n* (%)	1403 (57%)	157 (11%)	
	Yes, *n* (%)	1054 (43%)	168 (16%)	1.5 (1.19–1.9) *

* given a hand or kiss.

Table S2. Risk Factors associated with COVID-19 positivity confirmed by PCR test.

Covariates	Crude OR (95% CI)	Adjusted OR (95% CI)	*p*-Value
Gender			
Male	Ref	Ref	
Female	1.19 (0.91,1.55)	1.14 (0.87,1.49)	0.348
Age			
0–19 years,	Ref	Ref	
20–34 years,	1.23 (0.8,1.9)	1.2 (0.77,1.88)	0.419
35–49 years,	1.45 (0.94,2.23)	1.46 (0.94,2.27)	0.091
50–64 years,	1.26 (0.79,2.01)	1.33 (0.82,2.15)	0.245
65+ years,	1.58 (0.95,2.63)	1.73 (1.02,2.93)	**0.040**
Enough resources for living			
No	Ref	Ref	
Yes	0.7 (0.54,0.91)	0.74 (0.56,0.98)	**0.033 ***
Means of Transport to work			

Other(Foot/Private transport)<br>Public(BUS/Taxi)	Ref<br>1.66 (1.27,2.15)	Ref<br>1.58 (1.2,2.07)	**<0.001 ***
Being to a funeral(7 days prior)<br>No<br>Yes	Ref<br>2.33 (1.21,4.48)	Ref<br>2.17 (1.1,4.29)	**0.026 ***
Being in contact with someone outside your home with flu-like symptoms, No<br>Yes	Ref<br>1.58 (1.13,2.21)	Ref<br>1.55 (1.09,2.19)	**0.014 ***
Has anyone in your house had covid-19?, No<br>Yes	Ref<br>5.63 (3.78,8.38)	Ref<br>5.68 (3.73,8.65)	**<0.001 ***
Had a kiss within last week or today?<br>No kiss<br>Yes	Ref<br>1.66 (1.28–2.15)	Ref<br>1.8 (1.37–2.36)	**<0.001 ***
No of people in house<br>Living alone<br>2–4 people<br>5–6 people<br>>6 people	Ref<br>1.18 (0.76,1.84)<br>1.14 (0.71,1.84)<br>2.44 (1.42,4.2)	Ref<br>1.18 (0.75,1.87)<br>1.13 (0.69,1.87)<br>2.22 (1.25,3.95)	0.473<br>0.621<br>**0.006**
